# Modulation of ADAR mRNA expression in patients with congenital heart defects

**DOI:** 10.1371/journal.pone.0200968

**Published:** 2019-04-30

**Authors:** Faiza Altaf, Cornelia Vesely, Abdul Malik Sheikh, Rubab Munir, Syed Tahir Abbas Shah, Aamira Tariq

**Affiliations:** 1 Department of Biosciences, COMSATS University Islamabad, Islamabad Campus, Islamabad, Pakistan; 2 Center for Anatomy and Cell Biology, Medical University Vienna, Vienna, Austria; 3 Rawalpindi Institute of Cardiology, Rawalpindi, Pakistan; Northwestern University, UNITED STATES

## Abstract

Adenosine (A) to inosine (I) RNA editing is a hydrolytic deamination reaction catalyzed by the adenosine deaminase (ADAR) enzyme acting on double-stranded RNA. This posttranscriptional process diversifies a plethora of transcripts, including coding and noncoding RNAs. Interestingly, few studies have been carried out to determine the role of RNA editing in vascular disease. The aim of this study was to determine the potential role of ADARs in congenital heart disease. Strong downregulation of ADAR2 and increase in ADAR1 expression was observed in blood samples from congenital heart disease (CHD) patients. The decrease in expression of ADAR2 was in line with its downregulation in ventricular tissues of dilated cardiomyopathy patients. To further decipher the plausible regulatory pathway of ADAR2 with respect to heart physiology, miRNA profiling of ADAR2 was performed on tissues from ADAR2-/- mouse hearts. Downregulation of miRNAs (miR-29b, miR-405, and miR-19) associated with cardiomyopathy and cardiac fibrosis was observed. Moreover, the upregulation of miR-29b targets COL1A2 and IGF1, indicated that ADAR2 might be involved in cardiac myopathy. The ADAR2 target vascular development associated protein-coding gene filamin B (FLNB) was selected. The editing levels of FLNB were dramatically reduced in ADAR2^-/-^ mice; however, no observable changes in FLNB expression were noted in ADAR2^-/-^ mice compared to wild-type mice. This study proposes that sufficient ADAR2 enzyme activity might play a vital role in preventing cardiovascular defects.

## Introduction

Congenital heart disease (CHD) is defined as a structural or functional heart defect. It belongs to a heterogeneous group of diseases and can be classified anatomically, clinically, epidemiologically and developmentally [1–3]. The most common conditions associated with CHD among hospitalized patients are ventricular septal defect (VSD), Tetralogy of Fallot (TOF), patent ductus arteriosus (PDA), transposition of the great arteries (TGA), atrial septal defect (ASD) and atrioventricular septal defect (AVSD) [4]. A recent study indicated that, due to cardiac anomalies, 11% of Pakistani newborns die within the first postnatal month [5]. Genetic conditions and environmental factors, such as maternal diabetes and rubella, have been identified as factors leading to CHD defects [6].

The most common type of RNA editing in humans replacement of adenosine (A) with inosine (I) [7].ADARs perform this complex hydrolytic posttranscriptional deamination reaction. The ADAR family consists of three members, ADAR1, ADAR2 and ADAR3. ADAR1 and ADAR2 play active roles in the deamination of adenosine, while ADAR3 is nonfunctional [8]. Several studies have shown that the extent of RNA editing not only varies between individuals but also has high tissue specificity [9–11]. Approximately 2.5 million sites in the human transcriptome have been edited; a large majority of them are in double-stranded Alu elements, and the sites are mainly in introns and untranslated regions (UTRs) [8]. However, the functional consequences of a majority of the RNA editing events remain elusive.

RNA editing is known to modulate splicing, mRNA coding potential, and transcript stability and even to alter microRNA processing and targeting [7, 9, 10]. Initially majority of the RNA editing targets have been reported in the neuronal receptors such as AMPA and glutamate receptors [11, 12]. A recent study focusing on RNA editing events in six different tissues identified an average of 79,976 editing sites within heart tissue [8]. This implies that RNA editing might play a role in other tissues apart from the nervous system. Apart from protein coding targets different non-coding RNAs like microRNAs (miRNAs) are also edited by ADARs [13, 14]. ADARs modulate the processing and expression of miRNA by either editing [10, 14, 15] or interacting with dicer [16]. They also alter target specificity of miRNAs by editing the seed sequence [10]. miRNAs regulate diverse cellular physiology[17] and developmental processes [18]. In the cardiovascular system, miRNAs regulate diverse processes, such as cardiac remodeling, cardiac hypertrophy and fibrosis [19]. A significant decrease in ADAR2 RNA was observed in cyanotic congenital heart disease (CHD while expression of ADAR1,did not show a significant difference [20]. Another study demonstrated an increase in ADAR1 mediated RNA editing of the CTSS transcript is increased in hypoxic or proinflammatory conditions as well as in patients with clinical or subclinical vascular damage[21]. CTSS plays a role in vascular inflammatory processes.[21]. Moreover, decrease in ADAR2 mediated editing of another actin-binding protein, filamin A (FLNA), have been linked to cardiovascular disease and reduced systolic output [22]. Thus, these studies demonstrate that ADARs might be contributing towards heart disease.

The current study determined the RNA levels of ADAR1 and ADAR2 in congenital heart disease patients. The relative gene expression of a crucial angiogenesis-related transcription factor, FOXP1, was found to be downregulated in CHD cases. A dramatic decrease in ADAR2 mRNA levels and an opposing upregulation of ADAR1 were also found. To further explore the role of ADAR2 in heart physiology, ADAR2 knockout mouse heart tissue was used. Although previously documented, no strong anomalies in heart physiology were observed [23].

## Materials and methods

### Collection of samples

This study was initiated after receiving approval from the ethics committees of both COMSATS Institute of Information Technology (CIIT) (No./Bio/ERB/15/75) and the collaborating hospital, the Rawalpindi Institute of Cardiology (RIC). Patient echocardiography reports were consulted to confirm the presence of congenital heart defects. Blood samples from 35 patients with different defects from the Rawalpindi Institute of Cardiology (RIC) were collected before surgery and stored on ice during transportation. The samples were segregated on the basis of age (3 months– 16 years) and sex. Thirteen control samples were collected from healthy individuals using the same parameters. Interviews were conducted using specified questionnaires that gathered information on age, sex, medication and family history. The details of these persons are provided in S1 and S2 Tables in accordance with the Ethical Review Board (ERB) approval.

### cDNA synthesis

Five milliliters of whole blood was collected from each patient and stored in ethylenediaminetetraacetic acid (EDTA) test tubes. To avoid RNA degradation, the blood was kept at 4°C for up to 24 hours between sample collection and RNA extraction. RNA was isolated from peripheral blood mononuclear cells with TRIzol Reagent (Invitrogen, Germany) according to the manufacturer’s instructions. The optical density of the RNA was measured immediately following extraction. RNA samples showing 260/280 ratios below 1.8 or above 2.0 were not used in further analyses. Complementary DNA (cDNA) was synthesized from one microgram of RNA using a RevertAid First Strand cDNA Synthesis Kit (Thermo Scientific, USA). A negative control, termed minus reverse transcriptase (–RT), that lacked reverse transcriptase was prepared for each of the samples.

### Real-time PCR

The relative mRNA expression of genes was examined using quantitative PCR with gene-specific primer sets (IDT, USA and Macrogen, South Korea); the TUB1 gene was used as an internal control. 5X HOT FIREPol EvaGreen qPCR Mix Plus master mix (ROX) (Solis BioDyne, Tartu, Estonia) was used for qPCR. The sequences of the genes (ADAR1 p110, ADAR1 p150, ADAR2, FOXP1, FLNB, COL1A2, and IGF1) were obtained from Ensemble, and primers were synthesized with the Integrated DNA Technologies Primer Quest Tool. The primers are listed in S1 Table.

### Statistical analysis

Statistical analyses were performed with GraphPad Prism 7.0b. For expression data, the target gene (ADAR1 p110, ADAR1 p150, ADAR2, FOXP1, FLNB, COL1A2, and IGF1) Ct values were normalized to the control gene (TUB1) Ct value. Statistical significance was determined using the Mann-Whitney U test, and P< 0.05 was considered to indicate significance.

### Phenotype of ADAR2-/- mice

Adar2^−/−^ mice were a kind gift from Peter Seeburg. These transgenic mice were bred on an SV129 background. As ADAR2 deficiency leads to early postnatal lethality, the mice were rescued with a pre-edited Gria2 receptor (Gria2R/R) [24, 25]. The mice were bred at the Vienna BioCenter facility animal house. Gria2R/R; ADAR2^+/−^ mice were intercrossed. The resulting sibling female offspring of genotype Gria2R/R;Adar2^−/−^ and Gria2R/R;ADAR2^+/+^ were euthanized at the age of postnatal day 6 (P6) by cervical dislocation. The whole hearts were dissected and subsequently used for RNA preparation from three biological replicates [24, 26]. These mice displayed phenotypic features described previously [23].

### RNA extraction and microRNA cloning

RNA was extracted from the whole hearts of euthanized postnatal day 6 (P6) female mice (Adar2^-/-^Gria R/R and Adar2^+/+^, Gria R/R siblings) using TriFast (Peqlab, Erlangen, Germany). Generation of libraries for small RNA next-generation sequencing (NGS) was performed according to a method described previously [15].

Quantification of the complete library was performed using a Bioanalyzer and a qPCR NGS Library Quantification Kit (both from Agilent Technologies). Cluster generation and sequencing were carried out using an Illumina HiSeq 2000 system. After single-end sequencing at a read length of 50 base pairs, the adaptor sequences were removed using Cutadapt (http://code.google.com/p/cutadapt/). At least 200,000 reads per sample were generated and mapped to the genome using Bowtie [27]. The differential expression of microRNAs was analyzed using DESeq [28]. Only microRNAs with a P-value smaller than 0.1 were considered.

### Sequencing and clipping of reads

The completed libraries were quantified with an Agilent Bioanalyzer dsDNA 1000 Assay Kit and an Agilent qPCR NGS Library Quantification Kit. Cluster generation and sequencing were carried out using an Illumina Genome Analyzer IIx System according to the manufacturer's guidelines. Illumina sequencing was performed at the Vienna BioCenter Core Facility Next Generation Sequencing (VBCF NGS) Unit (csf.ac.at). After sequencing at a read length of 36 base pairs, the adaptor sequences were removed using Cutadapt [29]. The RNA sequencing data are available in the GEO database (GSE122397).

### Mapping to mature microRNA sequences

Mapping of the clipped reads to mature microRNA sequences was performed as described previously. Mapping was performed using the NextGenMap tool, restricting the mapped reads to have at least 90% identity (# differences/alignment length) [30]. Two microRNA target genes, COL1A2 and insulin-like growth factor-1 (IGF1), were amplified using the primers listed in S4 Table.

## Results

### ADAR2 expression is reduced in CHD patients

**Fig 1:**
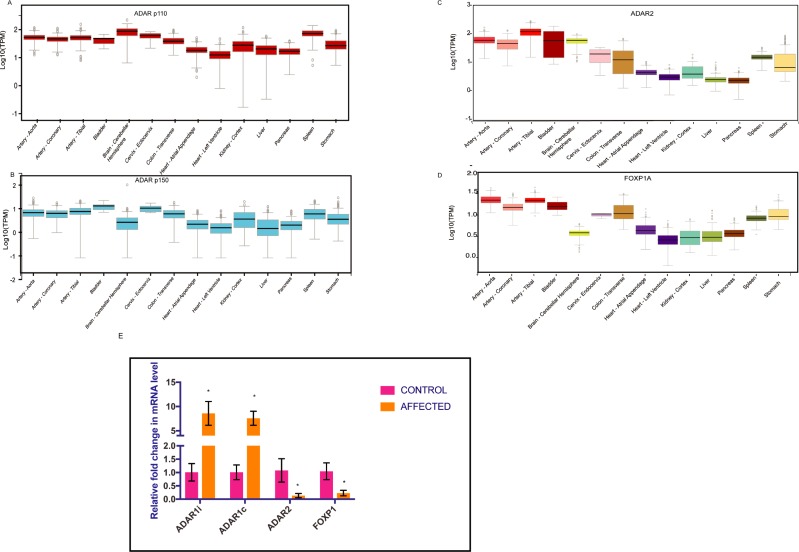
ADAR2 expression is reduced in CHD patients. **(**A) ADAR1 p110 is strongly expressed in brain cerebral hemispheres as compared to left heart ventricle. The Y-axis represented the log10 of the transcript per million (TPM) value. The whiskers represent the maximum and minimum values. The median is represented as a black line in the box. (B) The expression of ADAR1 p150 is strongest in the bladder, followed by the cervix and tibial artery. ADAR1p150 expression is slightly higher in the left heart ventricle as compared to cerebral hemisphere. The whiskers represent the maximum and minimum values. The median is represented as a black line in the box. (C) ADAR2 expression is strongest in the tibial artery, followed by the bladder, stomach aorta and coronary artery. The whiskers represent the maximum and minimum values. The median is represented as a black line in the box. (D) FOXP1 expression is strongest in the aorta, followed by the tibial artery and bladder. The whiskers represent the maximum and minimum values. The median is represented as a black line in the box. (E) Bar graph showing the relative RNA levels of ADAR1, ADAR2 and FOXP1 in patients compared to controls. ADAR2 and FOXP1 showed significantly reduced expression at the RNA level, whereas both isoforms of ADAR1 (ADAR1 p110 (ADAR1c) and p150 (ADAR1i)) showed upregulation in CHD patients. The error bars indicate the mean +/-SD. The statistical significance was determined by the Mann–Whitney U test *P < 0.05 (versus control).

RNA was extracted from blood samples taken from 35 patients diagnosed with congenital heart disease and from 13 otherwise healthy individuals, which were used as controls. Most of the patients had VSD. Initially, ADARs were thought to play an important role in the nervous system, as most of the editing targets, such as glutamate and AMPA receptors, were found in the brain [10, 11, 31, 32]. Recent studies indicate that ADARs might contribute towards cardiovascular disease [21, 22]. GTEX differential expression analysis of different tissues showed higher expression of the ADAR1 p110 in the nervous system, whereas ADAR1 p150 RNA level was higher in the vascular system (Fig 1A & 1B). ADAR1 p110 was strongly expressed in the brain cerebral hemispheres, followed by the spleen and tibial artery. In the Fig 1, the whiskers represent the maximum and minimum values. The median is represented as a black line in the box. GTEX gene expression analysis of different tissues showed higher expression of ADAR2 and FOXP1 vascular system compared to nervous system tissues (Fig 1C & 1D). As a control for CHD, we selected a member of the forkhead box family of transcription factors, FOXP1. FOXP1 plays a critical role in mouse and human heart development, and high expression has been observed during cardiomyocyte proliferation [33]. qPCR analysis showed a significant decline in ADAR2 expression in CHD patients compared to controls (Fig 1E). However, the expression of both ADAR1 isoforms (p110 and p150) was significantly increased in CHD patients This elevated ADAR1 expression is consistent with recent findings demonstrating that ADAR1 expression is elevated in patients undergoing carotid endarterectomy [21]. Of the three genes, FOXP1 and ADAR2 were downregulated in patients (Fig 1E), indicating their expression modulation with regard to heart disease.

### Heart defect-specific function

**Fig 2:**
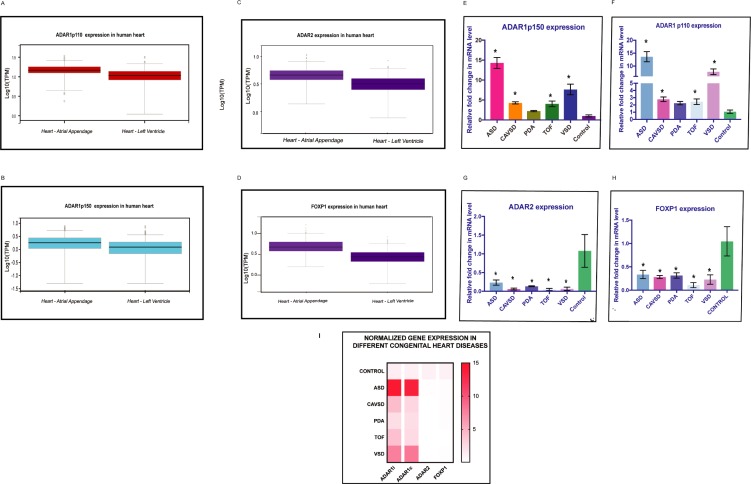
Heart defect-specific function. (A& B) ADAR1 p110 and ADAR1 p150 showed higher expression in the atrial appendage than in the left ventricle. The Y-axis represented the log10 of the transcript per million (TPM) value. The expression of ADAR1 p150 (log_10_TPM 0.5) is lower in heart atrium as compared to ADARp110 (log_10_TPM 1.0). The whiskers represent the maximum and minimum values. The median is represented as a black line in the box. This indicated that ADAR1 isoforms express strongly in the heart atrium as compared to ventricles. (C&D) ADAR2 and FOXP1 showed higher expression in the atrial appendage than in the left ventricle (log_10_TPM 0.7). The expression of ADAR2 and FOXP1 is lower as compared to ADAR1. The whiskers represent the maximum and minimum values. The median is represented as a black line in the box. (E& F) Bar graph showing upregulation of ADAR1 p110 (ADAR1c) and p150 (ADAR1i) in different CHD cases. Both isoforms are significantly upregulated in ASD and VSD. This indicated that both ADAR1 isoforms have potential role in atrial heart tissue. However, a 3- to 4-fold increase in RNA levels was found in AVSD and TOF cases compared to controls. This implies that ADAR1 isoforms are upregulated in response to cardiovascular defects. The PDA cases did not show a significant difference from controls. The error bars indicate the mean +/-SD. *P < 0.05 (versus control), as determined by the Mann–Whitney U test. (G) Bar graph showing a strong decline in ADAR2 expression, particularly in AVSD, TOF and VSD, in CHD cases compared to controls. This indicated that ADAR2 might have a potential role in both heart atrium and ventricular tissue. ADAR2 is significantly downregulated in all CHD cases. Error bars indicate the mean +/-SD. *P < 0.05 (versus control), as determined by the Mann–Whitney U test. (H). Bar graph showing reduced expression of FOXP1 in all CHD cases signifying its importance in cardiovascular development. The strongest decrease was observed in the TOF samples. *P < 0.05 (versus control), as determined by the Mann–Whitney U test. The error bars indicate the mean +/-SD. (I). Heat map showing the normalized expression levels of ADAR1 p150 (ADAR1i), ADAR1 p110 (ADAR1c), ADAR2 and FOXP1 in different CHD cases compared to controls. The expression values are normalized with the housekeeping gene TUB1. The red color indicates increase in expression whereas the lighter red represents decrease in expression as compared to control.

The differential expression of ADARs in the vascular tissues has further intrigued us to determine their expression in heart tissues. The expression analysis was compared between the atrium and ventricular tissues of the heart. GTEX expression analysis of heart tissue showed higher levels of ADAR1 p110 compared to p150 in the atrium. However, ADAR1 p150 showed similar expression in both atrial and ventricular tissue (Fig 2A & 2B). In addition, the expression of ADAR2 and FOXP1 was also high in the atrium compared to the ventricle (Fig 2C & 2D). The high atrial specific expression of ADARs was further correlated with the expression pattern of the three selected genes with the heart defect. The strongest upregulation was observed of both ADAR1 isoforms (p150 & p110) isoforms in ASD, followed by VSD. However, the results also showed an approximately 3-fold increase in the expression of these isoforms in patients with TOF and AVSD compared to controls (Fig 2E & 2F). In contrast, the ADAR2 mRNA level was decreased in AVSD, TOF and VSD patients (Fig 2G). The FOXP1 mRNA levels were decreased significantly in TOF patient samples compared to control samples as it is an important transcriptional regulator during cardiovascular development (Fig 2H). Thus, the high expression of both the ADAR1 isoforms in ASD implies that they might have a critical function in the atrium. The strong downregulation of ADAR2 indicated that it might play a role in atrium and ventricle development.

The marked decrease in ADAR2 expression in CHD patients was further investigated in ADAR2^-/-^ mouse hearts. ADAR2 mediated editing occurs both in coding and non-coding genes[34] Non-coding RNAs such as miRNAs play a significant role in heart development [35, 36].The small RNA sequencing of ADAR2^-/-^ mouse heart samples was performed in biological triplicates. No upregulated miRNAs were observed in the absence of ADAR2 editing activity. However, a consistent ~2-fold decrease in miR-29b levels was consistently observed at the P6 stage (GSE122397) in ADAR2^-/-^ mouse hearts compared to wild-type mouse hearts. miR-29b inhibits cardiac fibrosis induced by angiotensin II through the TGF-β/Smad3 pathway[36]. The deregulation of miR-29b can induce cardiac fibrosis [36]. Other miRNAs like miR-451b, miR-451a, and miR-19b (Table 1) showed ~ 1.5-fold downregulation. Apart from these, various members of the let-7 family also showed downregulation of approximately 1- to 1.5-fold in ADAR2 knockout mouse hearts (Table 1). Aberrant expression of the let-7 family has been linked to a variety of cardiovascular diseases, including fibrosis, hypertrophy, dilated cardiomyopathy (DM), myocardial infarction (MI), atherosclerosis and hypertension [37]. This reduction in the regulation of ADAR2 microRNAs points to the existence of a potential regulatory mechanism mediated by ADAR2 in cardiac development and physiology.

**Table 1:** miRNAs significantly downregulated in ADAR2^-/-^ mouse heart tissue as compared to wild-type mouse heart tissue in three biological replicates.

No.	miRNA	Log2 fold change	P value	Reference
1	miR-29b	-1.97	0.001599	[34]
2	miR-451b	-1.51	0.005	[37]
3	miR-451a	-1.51	0.005	[37]
4	miR-19b1	-1.48	0.015	[34]
5	let7c-2	-1.47	0.0017	[13]
6	let7c-1	-1.39	0.003	[13]
7	let7-i	-1.23	0.01	[13]
8	let7-b	-1.23	0.01	[13]
9	miR-382	-1.21	0.04	
10	miR-26a	-1.19	0.03	
11	miR-378	-1.19	0.011	[14]
12	miR-378a	-1.14	0.019	[14]
13	miR-130a	-1.09	0.024	[38]

Table 1 shows the 1- to 1.9-fold-downregulated miRNAs obtained by RNA sequencing analysis of the noncoding RNA dataset GSE122397.

**Fig 3:**
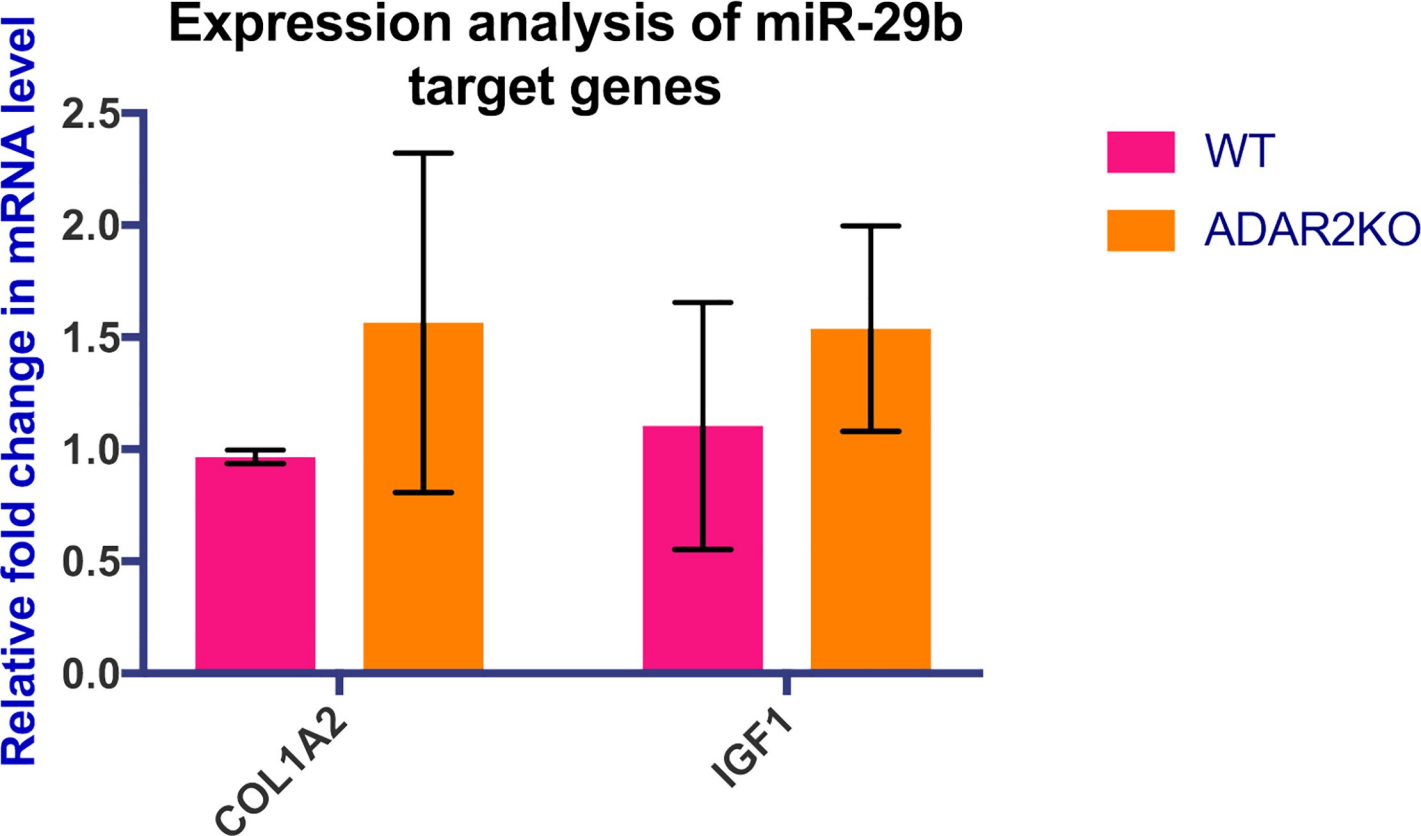
Expression analysis of the miR-29b target genes COL1A2 and IGF1 from ADAR2-KO mouse hearts in three different biological replicates. COL1A2 and IGF1 are cardiac hypertrophy and fibrosis related miR-29b target genes. miR-29b has been found downregulated ~2.0 fold in the absence of ADAR2. Bar graph showing slight but not significant changes in the expression of these genes. The error bars indicate the mean +/-SD. *P < 0.05 (versus control), as determined by the Mann–Whitney U test.

miR-29 family functions as downregulators of genes involved in cardiac fibrosis and extra cellular matrix production [19, 39]. The impact of the downregulation of miR-29b was determined on its target genes. Two genes Col1A2 and IGF1 were selected based on their role in cardiac fibrosis and hypertrophy. The upregulation of Col1A2 as a result of the miR-29b downregulation has been observed in the myocardial infarcted region [40]. Deficiency of IGF1 alleviates hypertrophic markers and mitigates cardiac hypertrophic remodelling [41]. qPCR analysis of two target genes, Col1A2 and IGF1, showed that these genes exhibited slight upregulation in knockout mouse hearts. This mild increase in Col1A2 and IGF1 can be due to the observed 2 fold decrease in miR-29b expression (Fig 3).

### Filamins and cardiac defects

**Fig 4:**
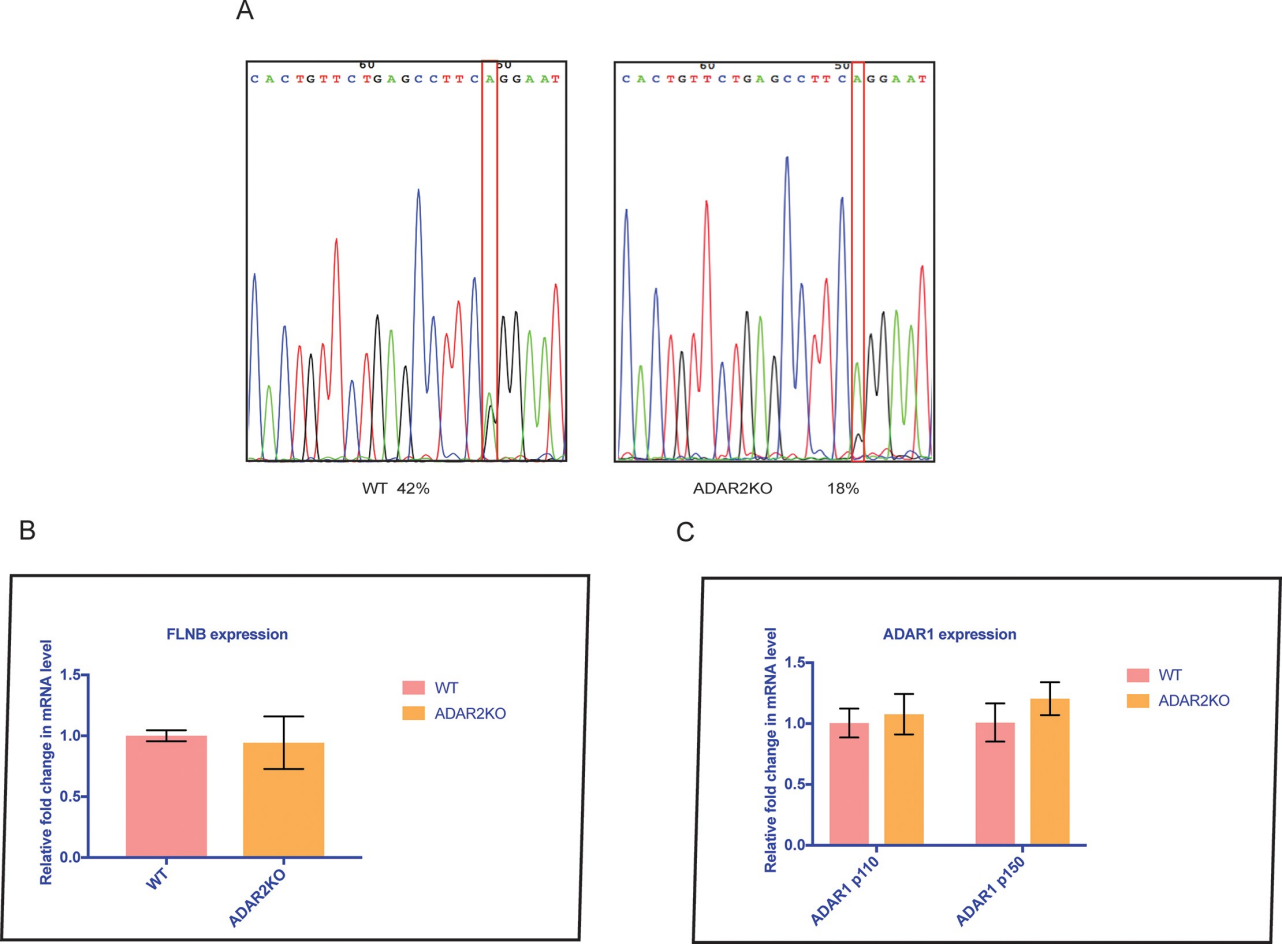
FLNB editing and ADAR1 expression in ADAR2-KO mice. **(A)** Electropherogram showing a 24% decrease in editing in ADAR2-KO compared to WT mouse tissues. This indicated that FLNB is primarily edited at the Q/R site by ADAR2. (B) **Expression analysis of FLNB.** The impact of FLNB editing on its expression was evaluated by semi-quantitative real time PCR. The bar graph showed that there was no significant difference in the expression of FLNB in the hearts of ADAR2^-/-^ mice compared to those of WT mice. This implies that editing of FLNB did not alter its expression. The error bars indicate the mean +/-SD. (C) **ADAR1 isoform expression in ADAR2**^**-/-**^ **mouse tissue**. The upregulation of ADAR1 in CHD is either because of the absence of ADAR2 or CHD occurrence. Therefore expression of ADAR1 isoforms was determined in the ADAR2KO mouse heart tissues. The bar graph showed no significant difference in the expression of the ADAR1 isoforms (p110 and p150) in the hearts of ADAR2-KO mice compared to those of WT mice. This implies that the upregulation of ADAR1 is due to CHD. The error bars indicate the mean +/-SD.

Actin-binding proteins such as FLNA and FLNB play important roles in the development of the vascular system [42]. ADAR2-mediated FLNA and FLNB RNA editing occurs at the same position, leading to changes in glutamine (Q) to arginine (R) at the protein level [43]. Impaired FLNA editing has been linked to arterial remodeling, thickening of the left ventricular wall, and increased phosphorylation of the myosin light chain[22]. FLNB showed high expression in vascular tissues [44]. High RNA editing of FLNB has been observed in the cardiovascular system (62% in the heart and 66% in the aorta) and musculoskeletal system. [44]. However, in ADAR2^-/-^ mouse hearts compared to wild-type mouse hearts FLNB editing was reduced by 24% (Fig 4). We propose that the elevated expression of FLNB and the decline of editing in ADAR2^-/-^ mouse heart tissues might be associated with the cardiovascular disease similar to FLNA. Since the editing of FLNB was correlated with FLNB expression in the mouse cerebral cortex [44] therefore, the mRNA level of FLNB in ADAR2^-/-^ hearts was also determined. No significant difference was observed in FLNB mRNA levels between ADAR2^-/-^ mouse heart tissues and wild-type mouse heart tissues (Fig 4). Thus, FLNB editing might have a heart-specific function independent of its expression.

### ADAR1 expression in ADAR2 KO mouse hearts

ADAR1 and ADAR2 both edit double stranded RNA. The elevated expression ofADAR1 in the absence of ADAR2 raised the possibility of a underlying compensatory mechanism for editing the target genes. Therefore, the expression of ADAR1 in ADAR2 KO mice was determined to rule out this possibility. ADAR1 mRNA levels remained unchanged in the absence of ADAR2 (Fig 4C). Therefore, we can conclude that the observed increase in ADAR1 was solely because of the CHD defect.

## Discussion

Adenosine deamination by ADARs is a posttranscriptional event that can diversify transcripts at both the sequence and structural levels. Deregulation of editing has been associated with a number of diseases [7, 20, 34]. The tissue-specific gene expression implies that ADAR1 p150, ADAR2 and FOXP1 might play critical roles in the vascular system. In this study, we observed a strong downregulation of ADAR2 and FOXP1 at the RNA level in PBMCs of congenital heart defect patients. The observed differences at the mRNA level might be due to differential expression patterns in atrium versus ventricular heart tissues. RNA sequencing analysis of left ventricular myocardium from 13 DCM individuals compared to 10 controls showed significant declines of ~1.5-fold and ~1.3-fold for ADAR2 and FOXP1, respectively[45] (GSE55296). However, no significant difference was seen at the ADAR1 mRNA level [45] (GSE55296). In addition, no significant deregulation was observed for the three candidate genes in ischemic cardiomyopathy cases [45] (GSE55296). This implies that the decrease in ADAR2 and FOXP1 is associated with dilated cardiomyopathy. However, ADAR1 mRNA levels were found to be significantly upregulated in dilated aortas (n = 35) compared to nondilated aortas (n = 8) [21]. A similar pattern of ADAR1 overexpression was observed in human atherosclerotic plaques [21]. In this study, a significant increase in ADAR1 p150 mRNA levels was observed in the ASD and VSD patient samples signifying the upregulation of ADAR1 in response to CHD.

Two candidate genes FOXP1 and ADAR2 showed a significant decline in CHD. FOXP1, which acts as a transcription factor, has a DNA-binding domain that binds to the consensus sequence 5’-TRTTKRY-3’ located in the promoters and enhancers of many genes [46]. FOXP1 is associated with cardiovascular development as the FOXP1-null embryos usually die at embryonic day E14.5 with defects in ventricular septation, myocardial maturation and outflow tract septation [47]. Moreover, FOXP1 maintains cardiac homeostasis by counteracting the hypertrophy-associated effects of NFAT3 [48]. Thus, FOXP1 serves as a regulator for maintaining cardiomyocyte size and proliferation [48]. Thus, the observed deregulation of FOXP1 in PBMCs and DCM tissues might be indicating disturbed cardiac homeostasis. The other candidate gene ADAR2, editing activity has been reported previously in the brain AMPA receptor; leading to seizures and, consequently, death [49]. However, a recent study has documented high ADAR2 activity in the vascular system compared to the nervous system [22]. ADAR2 edits a plethora of coding and non-coding transcripts. ADAR2 modulates the expression and processing of non-coding RNAs such as miRNAs [10, 14, 50]. miRNA profiling of ADAR2 knockout mouse heart tissues showed ~1.5- to 2-fold downregulation of miR-29b, miR-451, miR-19 and members of the let-7 family (Table 1). Most of these downregulated miRNAs like miR-29b, miR-19 and let-7 family are associated with cardiac fibrosis, hypertrophy and cardiomyocyte proliferation [51–53]. This miRNA deregulation in the absence of ADAR2 points towards its involvement in the regulation of the heart development and physiology. Since ADAR2 downregulation was associated with DCM we selected two cardiac hypertrophy and fibrosis associated genes (COL1A2 and IGF1) that are potential targets of miR-29b. COL1A2 functions in pathways associated with extracellular matrix receptor interactions and focal adhesion [54]. High amounts of collagen may lead to cardiac fibrosis [54] whereas increased expression of IGF1 have been reported previously in hypertrophic cardiomyopathy [55]. Increases in the expression of COL1A2 and IGF1 were observed on the corresponding decrease of miR-29b in he absence of ADAR2. This slight increase in COL1A2 and IGF1 in ADAR2^-/-^ mouse heart tissues indicated that ADAR2 is not the core factor related to cardiomyopathy. However, it might be one of the factors involved in this pathway.

Among the protein coding targets of ADAR, filamins have gained importance as they are involved in cardiovascular development [42]. Filamins are actin-binding proteins comprising three members: filamins A, B and C. Filamins not only bind to actin via their N-terminal domains but also serve as docking sites for different cytoplasmic proteins, membrane receptors and integrins [56]. Filamin A and filamin B are exclusively edited in a highly interactive region of the Q/R site by ADAR2 [43]. ADAR2-mediated RNA editing of FLNA is 100-fold higher in vascular tissue than in nervous tissues [22]. Mice with impaired FLNA editing show increased vascular contraction, cardiac remodeling and reduced systolic output [22]. However, no changes in steady-state levels or stability of FLNA was observed in the absence of FLNA editing signifying the importance of editing independent of FLNA expression [22]. The same editing site and the high editing activity of FLNB in the heart (62.3%) and skeletal muscle (68.5%) made it a suitable candidate for this study [44]. Moreover, FLNB deficiency in mice is associated with vascular defects. FLNB also plays a critical role in endothelial cell migration and angiogenesis [56]. In this study, the 24% decrease in FLNB editing was also accompanied with no change in FLNB at RNA level in ADAR2-KO mouse heart tissues.

These findings also imply that ADAR2 might have a cardioprotective function as it modulates the expression of miRNAs and their corresponding targets. Further studies are also needed to elucidate the role of edited FLNB in the heart. We propose that editing of FLNB might be altering its interaction with different docking proteins. Therefore, we can conclude that ADAR2 might not be the critical factor for cardiac development. However, the deregulation of miRNAs and FLNB editing might contribute to hypertrophic cardiomyopathy. Future studies, may help to decipher the link between cardiac defects and ADAR2 activity along with the impact of ADAR2 mediated editing on its target genes.

## Supporting information

S1 Table CHD patient data. Age and gender distribution of the congenital heart defect patients along with their medication status and family history.
(DOCX)

S2 Table Normal individual data. Age and gender distribution of the normal individuals.
(DOCX)

S3 Table Primer sets used for the qPCR analysis of gene expression. The sequences of the primers used in this study are given.
(DOCX)

S4 Table Primer sets used for the real-time PCR analysis of miR-29b target gene expression in the ADAR2-KO mouse heart tissues. (DOCX)
