# Supplementary material for: Modulation of ADAR mRNA expression in patients with congenital heart defects
Source: PLoS One. 2019 Apr 30;14(4):e0200968. doi: 10.1371/journal.pone.0200968 (PMC6490900; doi:10.1371/journal.pone.0200968)
Supplement: S1 Table — Age and gender distribution of the congenital heart defect patients along with their medication status and family history. (DOCX) [file pone.0200968.s001.docx]

| **Gender-CHD** | **Age-CHD** | **Heart Defect** | **CHD Medication** | **Supplements** | **History** |
| --- | --- | --- | --- | --- | --- |
| F | 6 | ASD | YES | NO | NO |
| F | 16 | ASD | YES | NO | YES |
| M | 8 | ASD | NO | NO | NO |
| M | 14 | CAVSD | YES | NO | NO |
| F | 4 | CAVSD | YES | NO | NO |
| M | 4 | CAVSD | YES | NO | NO |
| M | 13 | Other | YES | YES | NO |
| F | 10 | Other | YES | NO | NO |
| M | 1 | Other | YES | NO | NO |
| M | 16 | PDA | YES | NO | NO |
| F | 10 | PDA | YES | NO | NO |
| F | 11 | PDA | YES | NO | NO |
| M | 7 | TOF | YES | YES | NO |
| M | 1 | TOF | YES | YES | NO |
| M | 1 | TOF | NO | NO | NO |
| F | 15 | VSD | YES | NO | NO |
| F | 0.42 | VSD | NO | NO | YES |
| M | 10 | VSD | YES | NO | NO |
| M | 6 | VSD | YES | NO | NO |
| M | 7 | VSD | YES | NO | YES |
| M | 13 | VSD | YES | NO | YES |
| F | 4 | VSD | YES | NO | NO |
| F | 0.25 | VSD | YES | NO | NO |
| F | 3.66 | VSD | YES | YES | YES |
| M | 13 | VSD | YES | NO | NO |
| F | 7 | VSD | YES | NO | NO |
| F | 1.92 | VSD | YES | NO | NO |
| F | 4 | VSD | YES | YES | NO |
| M | 11 | VSD | YES | NO | NO |
| M | 4 | VSD | YES | NO | NO |
| M | 6 | VSD | YES | NO | NO |
| F | 6 | VSD | YES | YES | NO |
| M | 5 | VSD | YES | NO | NO |
| M | 16 | VSD | YES | NO | NO |
| F | 6 | VSD | YES | YES | NO |

**S1 Table** : Age and gender wise distribution of congenital heart defect patients
