# Supplementary material for: Modulation of ADAR mRNA expression in patients with congenital heart defects
Source: PLoS One. 2019 Apr 30;14(4):e0200968. doi: 10.1371/journal.pone.0200968 (PMC6490900; doi:10.1371/journal.pone.0200968)
Supplement: S2 Table — Age and gender distribution of the normal individuals. (DOCX) [file pone.0200968.s002.docx]

**S2 Table** : Age and gender wise distribution of normal individuals

| Age | Gender |
| --- | --- |
| 14 | M |
| 13 | F |
| 3 | M |
| 16 | M |
| 6 | M |
| 5 | M |
| 10 | M |
| 15 | M |
| 5 | M |
| 15 | M |
| 14 | F |
| 15 | F |
| 5 | F |
