# Supplementary material for: Modulation of ADAR mRNA expression in patients with congenital heart defects
Source: PLoS One. 2019 Apr 30;14(4):e0200968. doi: 10.1371/journal.pone.0200968 (PMC6490900; doi:10.1371/journal.pone.0200968)
Supplement: S3 Table — The sequences of the primers used in this study are given. (DOCX) [file pone.0200968.s003.docx]

**S3 Table : Gene specific primer sets used in PCR**

| Gene | Primers | Sequence |
| --- | --- | --- |
| *ADAR1* | Forward | 5΄-CATGGCTTTGCTGCTGAAT-3΄ |
|  | Reverse | 5΄-CTGCTTGCCTTGCTTCTTG -3΄ |
| *ADAR2* | Forward | 5΄-CGGAGATCCTTGCTCAGATTT-3΄ |
|  | Reverse | 5΄-GAGGTGCTGATGTACAGATGAA-3΄ |
| *FOXP1* | Forward | 5΄-TCTCCCAAGAGGAATGACAAAC-3΄ |
|  | Reverse | 5΄-CACTTGTTGCTGGAGGATCT-3΄ |
| *TUB1* | Forward | 5΄-GCAACCAGATCGGAGCCAAG-3΄ |
|  | Reverse | 5΄-CATCGTCCCAGGTTCTAGGTC-3΄ |
| *ADAR1p110* | Forward | 5΄-GGCAGCCTCCGGGTG-3΄ |
|  | Reverse | 5΄-CTGTCTGTGCTCATAGCCTTGA-3΄ |
| *ADAR1p150* | Forward | 5΄- CGGGCAATGCCTCGC -3΄ |
|  | Reverse | 5΄- AATGGATGGGTGTAGTATCCGC- 3΄ |
