# Supplementary material for: Modulation of ADAR mRNA expression in patients with congenital heart defects
Source: PLoS One. 2019 Apr 30;14(4):e0200968. doi: 10.1371/journal.pone.0200968 (PMC6490900; doi:10.1371/journal.pone.0200968)
Supplement: S4 Table — (DOCX) [file pone.0200968.s004.docx]

**S4 Table**: Primers used for the amplification of miR-29b target gene

| **Sr.No.** | **Primers** | **Sequence** |
| --- | --- | --- |
| 1 | F- MJ7159 | AAGGGTGCTACTGGACTCCC |
| 2 | R- MJ7160 | TTGTTACCGGATTCTCCTTTGG |
| 3 | F MJ7163 | CATGTGCTGGCAGTATAACCC |
| 4 | R MJ7164 | TCGGGAGGCTTGTTCTCCT |
